# Comparing Quality of Life in Breast Cancer Patients Who Underwent Mastectomy Versus Breast-Conserving Surgery: A Meta-Analysis

**DOI:** 10.3390/ijerph16244970

**Published:** 2019-12-06

**Authors:** Elvin T. Ng, Russell Z. Ang, Bach X. Tran, Cyrus S. Ho, Zhisong Zhang, Wanqiu Tan, Yu Bai, Min Zhang, Wilson W. Tam, Roger C. Ho

**Affiliations:** 1 Institute of Cognitive Neuroscience, Huaibei Normal University, Huaibei 235000, China; e0012298@u.nus.edu (E.T.N.); rsczzs@chnu.edu.cn (Z.Z.); zhangmin235000@163.com (M.Z.); pcmrhcm@nus.edu.sg (R.C.H.); 2 Yong Loo Lin School of Medicine, National University of Singapore, Singapore 119228, Singapore; russell.angzen@u.nus.edu; 3 Institute for Preventive Medicine and Public Health, Hanoi Medical University, Hanoi 100000, Vietnam; bach.ipmph@gmail.com; 4 Johns Hopkins Bloomberg School of Public Health, Baltimore, MD 21205, USA; 5 Department of Psychological Medicine, National University Hospital, Singapore 119228, Singapore; su_hui_ho@nuhs.edu.sg; 6 Institute for Health Innovation and Technology (iHealthtech), National University of Singapore, Singapore 117599, Singapore; cjytwq@163.com; 7 The China-Singapore (Chongqing) Demonstration Initiative on Strategic Connectivity Think, Tank, Chongqing 400043, China; 8 Alice Lee School of Nursing, National University of Singapore, Singapore 117597, Singapore; nurtwsw@nus.edu.sg; 9 Department of Psychological Medicine, Yong Loo Lin School of Medicine, National University of Singapore, Singapore 119228, Singapore; 10 Centre of Excellence in Behavioral Medicine, Nguyen Tat Thanh University (NTTU), Ho Chi Minh City 70000, Vietnam

**Keywords:** breast cancer, breast conserving, individualised patient profiles, patient stratification, phenotyping, quality of life, mastectomy, meta-analysis

## Abstract

The purpose of our study was to carry out a meta-analysis of current literature to determine whether total mastectomy and breast-conserving surgery induce different outcomes in quality of life, based on the breast-cancer-specific module of the European Organizaation for Research and Treatment of Cancer core questionnaire (EORTC QLQ-BR23) used postoperatively. A systematic literature search of PubMed and EMBASE was conducted. Observational clinical studies that compared the quality of life in different surgery groups and presented empirical findings were selected. Six studies met the inclusion criteria. Breast-conserving surgery has statistically significant better outcomes than mastectomy in three of the eight outcomes measured in the EORTC QLQ-BR23, namely body image (standard mean difference, SMD = 1.742, 95% CI 0.579–2.905, *p* = 0.003), future perspective (SMD = 0.606, 95% CI 0.075–1.138, *p* = 0.025) and systemic therapy side effects (SMD = −0.641, 95% CI 0.101–1.181, *p* = 0.020). Our study highlighted that breast-conserving surgery was preferred over mastectomy because breast-conserving surgery leads to better outcomes in body image, future perspectives and less systemic side effects.

## 1. Introduction

Breast cancer remains the most common female malignancy internationally [1]. Due to an increased emphasis on breast screening programmes, breast cancer is now being diagnosed in its earlier stages. This has led to more surgical options, specifically those that target better cosmetic outcomes. Currently, the two main forms of breast cancer surgery are mastectomy and breast-conserving surgery (BCS). BCS was introduced as an alternative to mastectomy in patients with lower stage cancers, as it is less aggressive and could offer better cosmetic outcomes with similar benefits as compared to mastectomy. Numerous studies have been done to compare the survival outcomes between the two surgical techniques [2,3,4], as well as the impact on psychological health such as depression [5]. Due to better survival outcomes for breast cancer patients over the past decades [6], an aspect that is becoming increasingly important is the quality of life (QoL) of these patients after surgery. QoL is defined by the World Health Organisation as “an individual’s perception of their position in life in the context of the culture and value systems in which they live and in relation to their goals, expectations, standards and concerns” [7]. Being a broad-ranging concept, QoL covers areas other than psychological health, such as physical health, personal beliefs and social relationships, and hence may be a more holistic representation of a patient. To evaluate QoL in cancer patients, the European Organisation for Research and Treatment of Cancer core questionnaire (EORTC QLQ-C30) was developed [8]. Specifically of interest in this paper is the breast cancer module (QLQ-BR23), which assesses outcomes such as body image, sexual functioning, sexual enjoyment, future perspective, systemic therapy side effects, breast symptoms, arm symptoms and upset with hair loss. The QLQ-BR23 has been validated in multiple countries as well as field-tested in cross-cultural studies [9,10,11]. Hence, the aim of this paper is to carry out a meta-analysis of current literature to compare postoperative outcomes in quality of life in breast cancer patients who underwent total mastectomy versus BCS, based on the EORTC QLQ-BR23.

## 2. Methods

### 2.1. Search Strategy

We conducted a literature search using the following databases: PubMed and EMBASE for English-language studies that were published between 1 January 2000 and 31 December 2018. Keywords included ‘‘breast carcinoma’’ OR ‘‘breast cancer’’ AND ‘‘quality of life’’ OR ‘‘EORTC’’ AND ‘‘mastectomy’’ AND ‘‘breast conserving surgery’’.

### 2.2. Inclusion and Exclusion Criteria

Studies were included if they met the following inclusion criteria: (1) observational clinical study; (2) focusing on postsurgery female breast cancer patients; (3) comparing quality of life between mastectomy and BCS using EORTC QLQ-BR23; (4) presenting empirical findings on the parameters of interest (mean, standard deviation, sample size). Exclusion criteria were as follows: (1) quality of life was assessed using tools other than EORTC QLQ-BR23; (2) parameters of interest were not included in the findings; (3) breast reconstruction was done.

### 2.3. Study Selection and Extracted Information

Study selection was done using the Preferred Reporting Items for Systematic Reviews and Meta-Analyses (PRISMA) guidelines [12]. Results from the database search were then downloaded into EndNote X9 (Clarivate Analytics, Philadelphia, PA, USA) for deduplication both electronically and manually. After applying inclusion and exclusion criteria to remove ineligible articles through title and abstract screening, the full texts of eligible articles were retrieved and subjected to a full review. Data extracted from the final pool of eligible articles were documented on a Microsoft Excel spreadsheet to include the following information: (1) first author’s last name, year of publication and country; (2) total sample size; (3) mean age of patients (including standard deviation and range where available); (4) mean time from surgery to survey (including range where available); (5) mean score and standard deviation for each of the eight measurable outcomes of QoL, and sample size in the mastectomy and BCS groups.

### 2.4. Statistical Analysis

Statistical analyses were conducted using the Comprehensive Meta-Analysis Version 2.0 programme (Biostat, Englewood, NJ, USA). Random effects model was adopted to calculate the aggregate prevalence and its 95% confidence interval (CI) [13]. Heterogeneity between studies was assessed and quantified via Cochran’s chi-square and I^2^ statistic, respectively [14]. A mixed-effects meta-regression was used to identify the contribution of different moderators to the heterogeneity [15]. The moderators examined were overall mean age of patients and mean years since surgery, as well as the respective mean age of patients and mean years since surgery in the mastectomy and BCS groups. The regression coefficients and the corresponding *p* values were used to assess the effect of the moderators [16]. Publication bias was evaluated using the modified Egger’s linear regression test. Significance was set at *p* < 0.05.

## 3. Results

**Figure 1:**
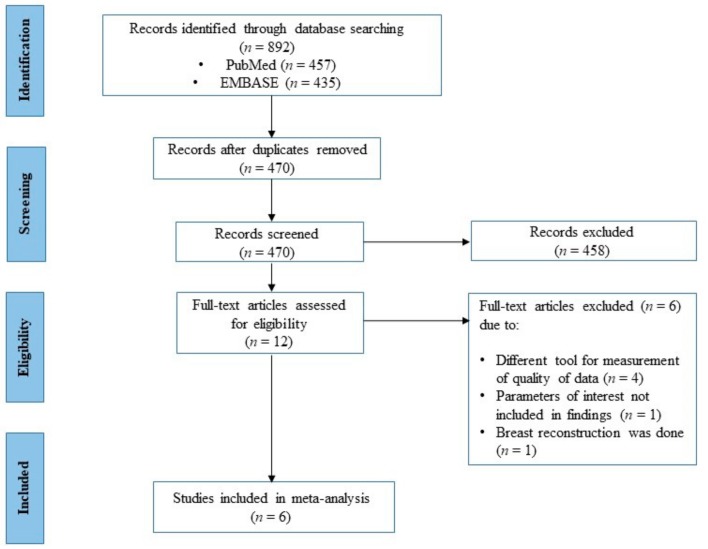
Process of study selection.

Eight hundred ninety-two studies were obtained from the literature search. After removing 422 duplicates, we excluded studies due to irrelevance to topic of interest (*n* = 440) and failure to meet inclusion criteria (*n* = 18). The remaining twelve full-text articles were assessed for eligibility, out of which six were excluded due to use of a different tool for measurement of quality of life (*n* = 4), parameters of interest not included in findings (*n* = 1), or breast reconstruction done (*n* = 1). A total of six studies were finally included in this meta-analysis. The process of study selection is summarised with the PRISMA flow diagram as depicted in Figure 1. All the final included studies were cross-sectional in design and had utilized the EORTC QLQ-BR23 questionnaire in comparing QoL in female breast cancer patients who had undergone mastectomy versus BCS.

### 3.1. Cohort Characteristics

The characteristics analysed included mean age and mean time since surgery (Table 1). The overall mean age of patients ranged from 51.35 to 67.33 years, and the mean time since surgery ranged from 4.08 to 6.34 years. All the patients were female who had undergone breast surgery due to breast cancer. None of them had breast reconstruction. The studies were conducted in two continents, namely Asia and Europe, with South Korea and Taiwan being the Asian studies, and Turkey, Netherlands and Germany making up the European studies.

### 3.2. Outcome Analysis

There are eight outcomes in the EORTC QLQ-BR23 questionnaire, which can be divided into two main groups, the functional and symptom domains. The functional domain comprises body image, sexual functioning, sexual enjoyment and future perspective, while the symptom domain comprises systemic therapy side effects, breast symptoms, arm symptoms and upset by hair loss. A higher score in functional domain is associated with a higher quality of life, while a higher score in the symptom domain is associated with a lower quality of life. While not all the studies analysed all eight outcomes, meta-analysis was still performed for each of the eight outcomes using the relevant studies. Three outcomes, namely body image, future perspective and systemic therapy side effects, were found to have statistically significant results.

#### 3.2.1. Body Image Outcome

**Figure 2:**
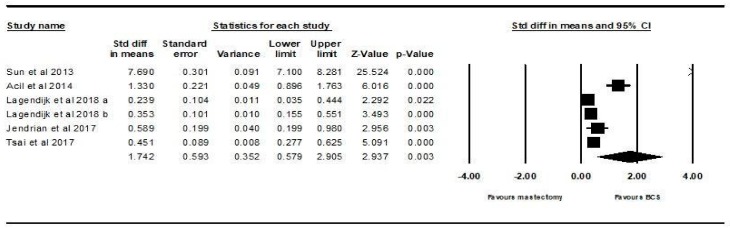
Forest plot showing the standardised mean difference of the body image score in patients who underwent mastectomy vs. BCS.

All six studies analysed body image. The random effects model showed that there is a statistically significant higher score, i.e., higher QoL, in patients who underwent BCS compared to those who underwent mastectomy (SMD = 1.742, 95% CI 0.579–2.905, *p* = 0.003). This is demonstrated using the forest plot as depicted in Figure 2.

#### 3.2.2. Future Perspective Outcome

**Figure 3:**
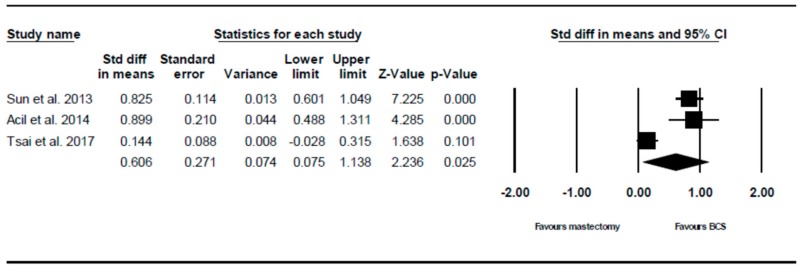
Forest plot showing the standardised mean difference of the future perspective score in patients who underwent mastectomy vs. BCS.

Three of the six studies analysed future perspective. The random effects model showed that there is a statistically significant higher score, i.e., higher QoL, in patients who underwent BCS compared to those who underwent mastectomy (SMD = 0.606, 95% CI 0.075–1.138, *p* = 0.025). This is demonstrated using the forest plot as depicted in Figure 3.

#### 3.2.3. Systemic Therapy Side Effects Outcome

**Figure 4:**
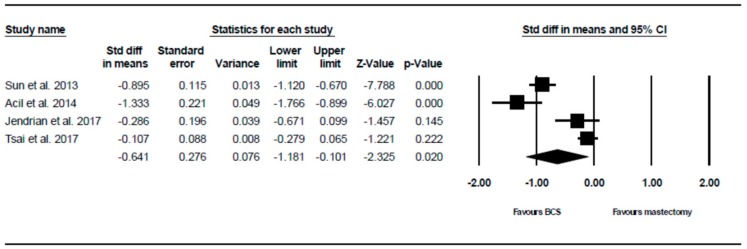
Forest plot showing the standardised mean difference of the systemic therapy side effects score in patients who underwent mastectomy vs. BCS.

Four of the six studies analysed systemic therapy side effects, The random effects model showed that there is a statistically significant lower score in patients i.e., higher QoL, who underwent BCS compared to those who underwent mastectomy (SMD = –0.641, 95% CI –1.181 to –0.101, *p* = 0.020). This is demonstrated using the forest plot as depicted in Figure 4.

The other five outcomes in the EORTC QLQ-BR23; namely sexual functioning, sexual enjoyment, upset by hair loss, arm symptoms and breast symptoms, did not yield statistically significant results. The forest plots for these five outcomes are depicted in Supplementary File (Figures S1–S5).

### 3.3. Meta-Regression and Publication Bias

Meta-regression was performed on two of the three significant outcomes, body image and systemic therapy side effects, using mean age and mean time since surgery as the covariates. The results can be found in Table 2 and Table 3**.** Meta-regression could not be performed on the future perspective outcome, because there were not enough studies for the number of moderators. Overall, there were no significant moderators found and no evidence of publication bias.

#### 3.3.1. Body Image

Mean age (B = −0.128, z = −1.14, *p* = 0.253) and mean time since surgery (B = −2.23, z = −1.49, *p* = 0.136) were nonsignificant moderators. There was no evidence of publication bias (intercept = 19.01, 95% CI −4.303 to 42.33, *t* = 2.26, df = 4, *p* = 0.0863).

#### 3.3.2. Systemic Therapy Side Effects

Mean age was a nonsignificant moderator (B = 0.0348, z = 0.76, *p* = 0.448). Mean time since surgery could not be analysed as a covariate as there were insufficient studies. There was no evidence of publication bias (intercept = 5.592, 95% CI −18.57 to 29.75, *t* = 0.996, df = 2, *p* = 0.424).

## 4. Discussion

### 4.1. Significant Results

In our meta-analysis, there were three outcomes in the QLQ-BR23 questionnaire that had statistically significantly better scores in the BCS group compared to the mastectomy group. The first significant outcome is body image. Multiple studies have shown the superiority of BCS in terms of postoperative body image when compared to mastectomy [26,27,28]. One possible reason is that mastectomy, being a more radical surgical technique, is associated with breast asymmetry and hence leads to a reduction in self-perceived attractiveness.

The second significant outcome is future perspective. We postulate that mastectomy inherently causes patients to feel that their future is compromised [29] as it is a radical surgery that is usually associated with higher-grade breast cancers and worse prognosis. On the other hand, BCS, which offers better aesthetic outcomes and is often performed for early breast cancer or benign breast tumours [30,31], may indirectly cause patients to be more optimistic about their long-term health.

The last significant outcome is systemic therapy side effects. A possible explanation for this could be that patients who underwent mastectomy usually have more advanced stage tumours which would subject them to more aggressive chemotherapy after the surgery, compared to those who underwent BCS. Therefore, systemic therapy side effects in the postmastectomy patients might be higher due to a larger proportion of these patients requiring a larger dose of chemotherapy and subsequently experience the side effects of dry mouth and taste alterations. Interestingly, this outcome was not expected as patients undergoing BCS are usually subjected to additional adjuvant therapy and hence should experience more side effects [32]. However, it is possible that women who had undergone BCS paid less attention to the side effects as they had stronger convictions regarding the benefits of surgery, including the preservation of breast appearance [33]. Consequently, they might underreport side effects such as dry mouth and taste alterations. On the other hand, women who had undergone mastectomy might feel more emotionally affected by the loss of a breast [34], and hence would be more sensitive to the systemic therapy side effects, introducing an element of over-reporting.

### 4.2. Nonsignificant Results

There were five outcomes that did not have any statistically significant differences in scores between the BCS and mastectomy groups. With regards to sexual enjoyment and sexual functioning, it is possible that both groups were affected similarly as sexual satisfaction is a complex and sophisticated process that is influenced by demographic, biological, psychological and sociocultural factors. Breast appearance is merely one of the factors amongst many others, such as occupation, duration of marriage, comorbidities and economic level [35]. In terms of breast and arm symptoms, there were also no significant differences between the two surgical techniques. This could be due to the differing thresholds for postoperative symptoms between patients. In fact, postoperative pain, which is one of the most common breast symptoms, has been shown to be not affected by different surgical techniques for breast cancer, but is instead increased by factors such as lower age, higher baseline anxiety and depression [36]. For arm morbidity, the movements most commonly affected are shoulder abduction, flexion and external rotation due to the site of surgery. However, there has not been any observed differences in pain on arm movement, as well as that of lymphedema (arm swelling), between BCS and mastectomy [37]. Lastly, both groups were equally upset by hair loss. We postulate that hair loss is a very distressing side effect as it is visibly noticeable both to patients as well as their friends and family, and hence affects both BCS and mastectomy patients equally.

### 4.3. Limitations

Several limitations have been identified in this meta-analysis. Firstly, QoL is only assessed by one questionnaire, the QLQ-BR23, which has intrinsic limitations. The validity of the QLQ-BR23 was evidenced by its ability to discriminate between subgroups of breast cancer patients known to differ in clinical status [38]. The subscales on body image, sexual functioning, arm symptoms and breast symptoms, and systemic therapy side effects were based on clinical perspectives but not to a broader understanding of these variables in psychology. While it is a relatively holistic representation, there are other factors that can affect QoL in breast cancer patients but are not addressed by QLQ-BR23, e.g., existential issues, spirituality and social relationships. The development of QLQ-BR23 was based on interviews held with breast cancer patients and medical specialists without referring to psychological theories of quality of life [39,40]. For example, the body-image subscale is comprised of physical attractiveness, femininity, difficulty to look at one’s body and dissatisfaction with body. Body image is a broad concept and the subscale was not based on cognitive and psychoanalytical theories. As the QLQ-BR23 could be filled out by the patients themselves with little or no assistance, the evaluation of QoL was subjective and information might be inaccurate. Furthermore, QoL is a dynamic indicator and can change over time, and hence a single survey done at a specific postoperative interval will not accurately reflect that. This meta-analysis only included studies published in English and this might limit the assessment of the accuracy and reliability of clinical conclusion to other populations. The validity and reliability of the QLQ-BR23 was established in other languages (e.g., Polish) [41]. Nevertheless, our findings are similar to some of the studies published in other languages. A Polish study found that patients treated with breast conserving surgery had a better score for body image [33]. Similarly, another Polish study reported that the type of surgical technique does not affect sexual satisfaction, which is similar to our findings [42]. Lastly, we only performed meta-regression on quantitative data such as age and time since surgery. Other possible moderators such as education and income levels were not provided in some of the studies we selected.

### 4.4. Future Research

The findings of this meta-analysis suggest future research direction. Due to the limitations of QLQ_BR23, future research adopting a holistic approach of QoL on breast cancer patients is required. Due to high heterogeneity of patients with breast cancer, the needs of patients, appropriate diagnostic approaches and optimal treatments depend on the individualised patient profile including biomarker panel [43], phenotyping based on circulating miRNA profiles [44] and detailed multiomic characterisation [45]. Future research can compare the QoL outcomes based on different interventions to treat breast cancer, driven by individual profiling. Furthermore, future QoL research should focus on specific groups, including pregnancy-associated breast cancer [46] and Flammer Syndrome phenotype, which involves the epigenetic predisposition of women at risk to form systemic hypoxic premetastatic niches that can be established long before breast malignancy is clinically manifested [47]. Flammer syndrome is known to be associated with particularly poor outcomes [47] and its impact on treatment and QoL is not well-studied.

## 5. Conclusions

Our meta-analysis suggests that breast-conserving surgery was preferred over mastectomy because breast-conserving surgery leads to better outcomes in body image, future perspectives and less systemic side effects. Larger prospective multicentre studies that assess QoL at multiple time intervals postoperatively and based on individualised treatment will be useful to determine the impact on QoL in the long run. This will enable breast cancer patients to be better informed when deciding treatment options.

## Figures and Tables

**Table 1:** Cohort characteristics of included studies.

Study	Year	Country	Total *n*	Age, Mean ± SD (Range)	Mean Time from Surgery to Survey (Range)
Sun [17]	2013	Korea	376	51.6 (28–70)	4.08 (2–8.67) years
Acil [18]	2014	Turkey	100	51.83 ± 9.26 (34–76)	NA
Lagendijk (a) [19]	2018	Netherlands	385	54.93 ± 8.77 ^a^	5 ^a^ years
Lagendijk (b) [20]	2018	Netherlands	419	51.35 ± 12.65 ^a^	6.34 ^a^ years
Jendrian [21]	2017	Germany	107	67.33 ± 9.40 ^a^ (36.4–83.8)	4.44 ^a^ (0.2–16) years
Tsai [22]	2017	Taiwan	544	52.8 ± 9.4	NA

SD, standard deviation; NA, not available. ^a^ Median, range and/or interquartile ranges were used as an estimate to calculate mean age and mean time since surgery [23,24,25].

**Table 2:** Results for meta-regression analysis (body image).

Predictor	No. of Studies Used	Univariate Coefficient	Z-Value	*p*-Value	Estimated Tau^2^
Mean age	6	−0.128	−1.14	0.253	2.38
Mean time since surgery (years)	4	−2.23	−1.49	0.136	6.57

**Table 3:** Results for meta-regression analysis (systemic therapy side effects).

Predictor	No. of Studies Used	Univariate Coefficient	Z-Value	*p*-Value	Estimated Tau^2^
Mean age	4	0.0348	0.760	0.448	0.334

## Supplementary Materials

The following are available online at https://www.mdpi.com/1660-4601/16/24/4970/s1: Figure S1: Forest plot showing the standardised mean difference of the sexual enjoyment score in patients who underwent mastectomy vs BCS (SMD = 0.213; 95% CI −0.265–0.691; *p* = 0.383), Figure S2: Forest plot showing the standardised mean difference of the sexual functioning score in patients who underwent mastectomy vs BCS (SMD = 0.025; 95% CI −0.378–0.427; *p* = 0.904), Figure S3: Forest plot showing the standardised mean difference of the upset with hair loss score in patients who underwent mastectomy vs BCS (SMD = −0.444; 95% CI −0.952–0.063; *p* = 0.086), Figure S4: Forest plot showing the standardised mean difference of the arm symptoms score in patients who underwent mastectomy vs BCS (SMD = −1.153; 95% CI −2.439–0.134; *p* = 0.079), Figure S5: Forest plot showing the standardised mean difference of the breast symptoms score in patients who underwent mastectomy vs BCS (SMD = −0.374; 95% CI −0.973–0.224; *p* = 0.220).
