# Supplementary material for: Comparing Quality of Life in Breast Cancer Patients Who Underwent Mastectomy Versus Breast-Conserving Surgery: A Meta-Analysis
Source: Int J Environ Res Public Health. 2019 Dec 6;16(24):4970. doi: 10.3390/ijerph16244970 (PMC6950729; doi:10.3390/ijerph16244970)
Supplement: Supplementary file 1 [file ijerph-16-04970-s001.pdf]

Supplementary file

Study name	Std diff in means	Standard error	Variance	Lower limit	Upper limit	Z-Value	p-Value
Sun et al 2013	-0.394	0.111	0.012	-0.612	-0.176	-3.546	0.000
Acil et al 2014	0.536	0.204	0.041	0.137	0.935	2.633	0.008
Jendrian et al 2017	0.319	0.196	0.039	-0.066	0.704	1.625	0.104
Tsai et al 2017	0.433	0.089	0.008	0.260	0.607	4.896	0.000
	0.213	0.244	0.060	-0.265	0.691	0.872	0.383

(Statistics for each study)

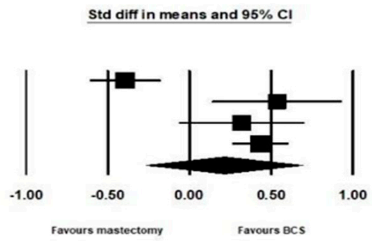

Figure S1. Forest plot showing the standardised mean difference of the sexual enjoyment score in patients who underwent mastectomy vs BCS (SMD = 0.213; 95% CI −0.265–0.691; *p* = 0.383).

Study name	Std diff in means	Standard error	Variance	Lower limit	Upper limit	Z-Value	p-Value
Sun et al 2013	-0.974	0.116	0.013	-1.201	-0.748	-8.419	0.000
Acil et al 2014	0.556	0.204	0.042	0.157	0.956	2.729	0.006
Lagendijk et al 2018 a	0.159	0.104	0.011	-0.045	0.363	1.523	0.128
Lagendijk et al 2018 b	0.168	0.100	0.010	-0.029	0.365	1.675	0.094
Jendrian et al 2017	0.143	0.196	0.038	-0.240	0.526	0.731	0.465
Tsai et al 2017	0.155	0.088	0.008	-0.017	0.327	1.770	0.077
	0.025	0.205	0.042	-0.378	0.427	0.120	0.904

(Statistics for each study)

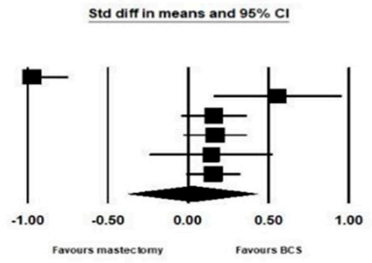

Figure S2. Forest plot showing the standardised mean difference of the sexual functioning score in patients who underwent mastectomy vs BCS (SMD = 0.025; 95% CI −0.378–0.427; *p* = 0.904).

Study name	Std diff in means	Standard error	Variance	Lower limit	Upper limit	Z-Value	p-Value
Sun et al 2013	-0.188	0.110	0.012	-0.404	0.029	-1.699	0.089
Acil et al 2014	-1.630	0.231	0.053	-2.082	-1.177	-7.060	0.000
Jendrian et al 2017	-0.047	0.195	0.038	-0.430	0.336	-0.240	0.811
Tsai et al 2017	-0.048	0.088	0.008	-0.220	0.123	-0.552	0.581
	-0.444	0.259	0.067	-0.952	0.063	-1.715	0.086

(Statistics for each study)

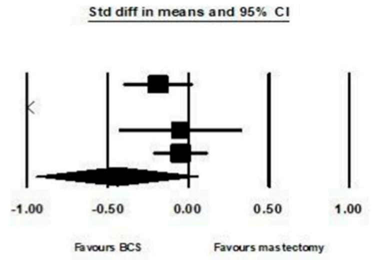

Figure S3. Forest plot showing the standardised mean difference of the upset with hair loss score in patients who underwent mastectomy vs BCS (SMD = −0.444; 95% CI −0.952–0.063; *p* = 0.086).

Study name	Std diff in means	Standard error	Variance	Lower limit	Upper limit	Z-Value	p-Value
Sun et al 2013	-4.457	0.196	0.039	-4.842	-4.072	-22.700	0.000
Acil et al 2014	-0.813	0.208	0.043	-1.221	-0.405	-3.908	0.000
Lagendijk et al 2018 a	-0.326	0.105	0.011	-0.531	-0.121	-3.112	0.002
Jendrian et al 2017	-0.158	0.196	0.038	-0.541	0.225	-0.808	0.419
Tsai et al 2017	-0.036	0.088	0.008	-0.207	0.136	-0.410	0.682
	-1.153	0.656	0.431	-2.439	0.134	-1.756	0.079

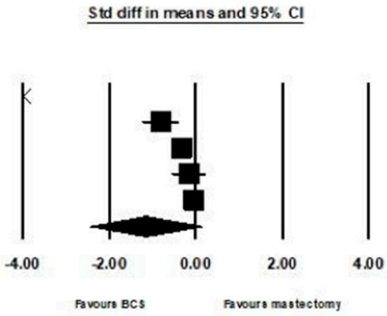

Figure S4. Forest plot showing the standardised mean difference of the arm symptoms score in patients who underwent mastectomy vs BCS (SMD = −1.153; 95% CI −2.439–0.134; $p = 0.079$).

Study name	Std diff in means	Standard error	Variance	Lower limit	Upper limit	Z-Value	p-Value
Sun et al 2013	-1.199	0.119	0.014	-1.431	-0.967	-10.116	0.000
Acil et al 2014	-0.959	0.211	0.045	-1.373	-0.545	-4.542	0.000
Lagendijk et al 2018 a	0.308	0.105	0.011	0.103	0.513	2.946	0.003
Jendrian et al 2017	-0.081	0.195	0.038	-0.463	0.302	-0.412	0.680
Tsai et al 2017	0.026	0.088	0.008	-0.145	0.198	0.300	0.764
	-0.374	0.305	0.093	-0.973	0.224	-1.227	0.220

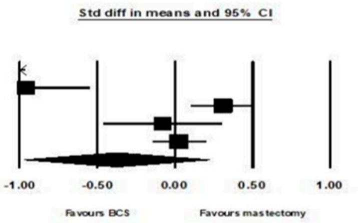

Figure S5. Forest plot showing the standardised mean difference of the breast symptoms score in patients who underwent mastectomy vs BCS (SMD = −0.374; 95% CI −0.973–0.224; $p = 0.220$).
